# Early clinical predictors of fulminant myocarditis in pediatric patients: a retrospective cohort study

**DOI:** 10.3389/fcvm.2025.1678220

**Published:** 2025-10-09

**Authors:** Jia Yuan, Ming Li, Li Ma, Zonglv Wu, Yujian Wu, Yuping Huang, Zongxiang Yin, Na Zhou

**Affiliations:** ^1^ Department of CICU, Guangzhou Women and Children’s Medical Center, Guangzhou Medical University, Guangzhou, Guangdong, China; ^2^ Department of Cardiac Surgery, Guangzhou Women and Children’s Medical Center, Guangzhou Medical University, Guangzhou, Guangdong, China; ^3^ Department of Ultrasound Diagnostics, Sunshine Union Hospital, Affiliated Teaching Hospital of Weifang Medical University, Weifang, Shangdong, China

**Keywords:** acute myocarditis, fulminant myocarditis, children, factors risk, clinical indicators

## Abstract

**Objective:**

Various clinical indicators can increase the likelihood of early identification of fulminant myocarditis, the identification of which is important for early treatment.

**Method:**

The medical records of all patients (*n* = 269) who were diagnosed with acute myocarditis between January 2014 and December 2023 were retrospectively analyzed. Patients were divided into two groups: the nonfulminant myocarditis group (*n* = 229) and the fulminant myocarditis group (*n* = 40). Baseline demographics, laboratory findings, electrocardiograms, echocardiograms, and treatment regimens were compared between the two groups via multifactorial analysis. A receiver operating characteristic (ROC) curve was used to explore the predictive value of related factors.

**Results:**

The median age of patients with fulminant myocarditis was significantly greater than that in the nonfulminant group (*P* = 0.015). The presenting symptoms at admission varied and included fever and respiratory, digestive, and circulatory symptoms. Among them, fever and hypotension were more common in the fulminant myocarditis group (*P* < 0.05), and vomiting was significantly more common in the nonfulminant myocarditis group (*P* = 0.017). Logistic regression analysis revealed that N-terminal pro-B-type natriuretic peptide (NT-proBNP), lactate (Lac), alanine aminotransferase (ALT), cardiac troponin I (cTnI), chest distress, and hypotension were early risk factors for fulminant myocarditis. ROC curve analysis demonstrated that NT-proBNP, ALT, cTnI, and Lac can serve as predictors for the early diagnosis of fulminant myocarditis. The optimal predictive values for these markers are 2,783.5 pg/ml for NT-pro BNP, 34.5 U/L for ALT, 0.2 µg/ml for cTnI, and 3.05 mmol/L for Lac.

**Conclusions:**

This study revealed that NT-proBNP, cTnI, ALT, and Lac can serve as predictive factors for the early identification of fulminant myocarditis. These findings emphasize the importance of early identification and timely diagnosis for improving the overall prognosis of patients.

## Introduction

Acute myocarditis in children can be caused by both infectious and noninfectious factors, with viral myocarditis being the most common cause (1–3). The clinical manifestations of acute myocarditis in children vary in severity. Children with mild myocarditis generally have a good prognosis. However, approximately 20% of children with fulminant myocarditis experience a sudden onset, leading to hemodynamic disorders and the need for circulatory auxiliary support (4). This condition can rapidly progress to refractory cardiogenic shock, lethal arrhythmic events, and even cardiac arrest, accounting for approximately 10%–38% of all acute myocarditis cases (5, 6). Currently, endocardial myocardial biopsy (EMB) remains the gold standard for diagnosing acute myocarditis. However, EMB is an invasive procedure that depends on the sampling site and has low sensitivity (7). More importantly, few institutions can perform this procedure and possess extensive relevant experience. As a result, it is difficult to widely promote this method in clinical practice (7). Previous studies have shown that LVEF, NT-proBNP, hypotension, and prolonged QRS are risk factors for identifying pediatric fulminant myocarditis in the early stage (5, 8). The aim of this study is to examine the early predictors of the collection of various clinical indicators can increase the likelihood of early identification of fulminant myocarditis, which is important for guiding early treatment.

## Method

### Study population

All patients admitted to Guangzhou Women and Children’s Medical Center, Guangzhou Medical University, with a diagnosis of acute myocarditis between January 2014 and December 2023 were retrospectively included in the analysis. The inclusion criteria were as follows: (1) age less than 18 years and (2) a diagnosis of acute myocarditis. According to the 2021 scientific statement of the American Heart Association (AHA), the typical diagnosis of acute myocarditis in children is ventricular dysfunction (with or without ventricular dilation), new-onset heart failure, and viral infection several weeks before onset (2). Currently, there are no clear diagnostic criteria for fulminant myocarditis in children; these criteria were based on the Chinese Medical Association Cardiovascular Society guidelines for adult fulminant myocarditis diagnosis and treatment, created with Chinese expert consensus. When acute myocarditis suddenly and rapidly progresses, severe heart failure, hypotension or cardiogenic shock soon appear, and the use of positive inotropic drugs, vascular active drugs or mechanical circulation adjuvant therapy can lead to a diagnosis of fulminant myocarditist (9). Thus, we classified acute myocarditis into fulminant myocarditis and nonfulminant myocarditis groups based on the severity of the condition. Ethical approval for the present study was granted by the Ethics Committee of Guangzhou Women and Children's Medical Center, Guangzhou Medical University (Approval No. 257A01). Informed consent was obtained from the parents or legal guardians.

### Clinical data collection

The following clinical data were collected: demographic information; hemodynamic parameters; cardiac rhythm; and laboratory findings, including white blood cell (WBC) counts; and C-reactive protein (CRP), aminotransferase (AST), alanine aminotransferase (ALT), creatine kinase-MB isoenzyme (CK-MB), cardiac troponin I (cTnI), serum creatinine (Scr), N-terminal pro-B-type natriuretic peptide (NT-proBNP), and lactate (Lac) levels measured at admission. Twelve-lead electrocardiogram (ECG) and echocardiogram examinations were performed on all patients. All admitted patients received treatments, including intravenous immunoglobulin (IVIG), corticosteroids, and vitamin C. Vasoactive drugs were used for those with hypotension. For patients whose conditions remained critical after treatment, extracorporeal membrane oxygenation (ECMO) support was initiated, and some patients received continuous renal replacement therapy (CRRT) when indicated.

### Statistical analysis

All the statistical analyses were performed using SPSS software, version 29.0, for Windows. Continuous variables are reported as the means ± SDs or medians and interquartile ranges, in line with a normal or non-normal distribution according to the Shapiro–Wilk normality test. Groups were compared via the unpaired Student's *t* test when normally distributed, whereas the Mann–Whitney *U* test was applied to those with a non-normal distribution. The Wilcoxon matched-paired signed-rank test was used to analyze paired data at different time points. Categorical variables were compared using Fisher's exact test. Factors with statistical significance in the univariate analysis and those considered clinically or previously significant were included in a logistic regression model for multivariate analysis. A receiver-operating characteristic (ROC) curve was used to explore the predictive value of related factors. A two-tailed *P* < 0.05 was considered statistically significant.

## Results

### Demographic features

A total of 269 patients were enrolled in this study. There were two groups: the nonfulminant myocarditis group (*n* = 229) and the fulminant myocarditis group (*n* = 40). The median age of patients with fulminant myocarditis was significantly greater than that of patients with nonfulminant myocarditis (*p* = 0.015). In the non-fulminant myocarditis group, age ranged from 3 months to 16 years, whereas in the fulminant myocarditis group it ranged from 1 year to 17 years. In this study, there were no neonatal patients. Age <3 years old, there were 79 patients. The presenting symptoms at admission varied and included fever and respiratory, digestive and circulatory symptoms. Among these patients, those with fever and hypotension were more likely to be in the fulminant myocarditis group (*p* < 0.05), and those with vomiting were significantly more likely to be in the nonfulminant myocarditis group (*p* = 0.017). Among all the patients, only 8 patients with fulminant myocarditis died (Table 1).

**Table 1:** Clinical presentations of pediatric patients with acute myocarditis.

Variable	Nonfulminant myocarditis (*n* = 229)	Fulminant myocarditis (*n* = 40)	*P*
Demographic data, *n* (%)			
Sex			0.05*
Male	130 (56.8)	16 (40)	
Female	99 (43.2)	24 (60)	
Age (years)	6 (3, 10)	7.5 (5.25, 10.75)	0.015*
Presenting symptoms, *n* (%)			
Fever	114 (49.8)	28 (70)	0.018*
Cough	96 (41.9)	13 (32.5)	0.263
Vomiting	39 (17)	1 (2.5)	0.017*
Abdominal pain	21 (9.2)	3 (7.5)	0.967
Chest pain	35 (15.3)	3 (7.5)	0.192
Chest distress	24 (10.5)	5 (12.5)	0.917
Palpitation	9 (3.9)	4 (10)	0.211
Hypotension	14 (6.1)	19 (47.5)	<0.001*
Prognosis, *n* (%)			
Death	0	8 (20)	<0.001*
Electrocardiograph, at admission, *n* (%)			
Low voltage	5 (2.2)	2 (5)	0.621
Ⅰ°A-V block	2 (0.9)	1 (2.5)	0.93
Ⅱ°A-V block	8 (3.5)	0	0.487
Ⅲ°A-V block	0	7 (17.5)	<0.001*
Supraventricular tachycardia	13 (5.7)	8 (20)	0.005*
Ventricular tachycardia	0	8 (20)	<0.001*
T-wave change	77 (33.6)	23 (57.5)	0.004*
Echocardiogram at admission, *n* (%)			
LVFS	34 (31, 37)	24 (19, 36.75)	<0.001*
LVEF	61 (58, 65)	47.5 (35, 62)	<0.001*
MR	61 (26.6)	23 (57.5)	<0.001*
TR	65 (28.4)	25 (62.5)	<0.001*
Pericardial effusion	21 (9.2)	12 (30)	<0.001*
Laboratory findings			
WBC (10^9^/L)	8.30 (6.07, 12)	10.32 (7.22, 12.65)	0.096
CRP (mg/L)	6.8 (0.84, 15)	11.94 (2.75, 21.3)	0.038*
Lac (mmol/L)	1.8 (1.27, 2.4)	3.3 (1.73, 6.25)	<0.001*
CK-MB (U/L)	26 (20, 48)	76.5 (32.5, 123.25)	<0.001*
NT-proBNP (pg/ml)	341.3 (115.59, 1,178.58)	9,614.5 (4,130.5, 28,954.81)	<0.001*
ALT (U/L)	24 (14, 43.5)	63 (31.25, 202.5)	<0.001*
AST (U/L)	42 (27, 61.5)	133.5 (75.75, 434.75)	<0.001*
Scr (umol/L)	32 (22, 43.5)	49 (35, 81)	<0.001*
cTnI (ug/L)	0.12 (0.06, 0.24)	0.48 (0.15, 1.23)	<0.001*
Treatment, *n* (%)			
IVIG	117 (51.1)	39 (97.2)	<0.001*
Corticosteroids	88 (38.4)	39 (97.2)	<0.001*
Vitamin C	154 (67.2)	34 (85)	0.038*
Vasoactive agent	14 (6.1)	40 (100)	<0.001*

A–V block, atrioventricular block; LVEF, left ventricular ejection fraction; LVFS, left ventricular fractional shortening; MR, mitral regurgitation; TR, tricuspid regurgitation; WBC, white blood cell count; CRP, fast C-reactive protein; Lac, lactate; CK-MB, creatine kinase isoenzyme; cTnI, cardiac troponin I; NT-proBNP, N-terminal B-type natriuretic peptide precursor; Scr, serum creatinine; AST, aspartate aminotransferase; ALT, alanine aminotransferase; IVIG, Intravenous immunoglobulin.

* *P* *<* 0.05.

### Electrocardiograms and echocardiograms

Approximately, 75.5% of patients had varying degrees of abnormal manifestations observed by electrocardiogram. Patients with fulminant myocarditis were significantly more likely to have severe arrhythmias with unstable circulation, including III°A–V block (*p* < 0.05), supraventricular tachycardia *(P* = 0.005), and ventricular tachycardia (*p* < 0.05). The left ventricular ejection fraction (LVEF) and left ventricular fractional shortening (LVFS) of patients with fulminant myocarditis were significantly lower than those of patients with nonfulminant myocarditis. The incidences of mitral regurgitation (MR), tricuspid regurgitation (TR), and pericardial effusion were significantly higher in patients with fulminant myocarditis (Table 1).

### Laboratory data

Table 1 summarizes the results of the initial laboratory tests conducted at admission. Compared to the nonfulminant myocarditis group, the fulminant myocarditis group had significantly increased CRP, CK-MB, NT-proBNP, cTnI, ALT, AST, and Lac levels at admission (*p* < 0.05).

### Early identification of risk factors for fulminant myocarditis

**Figure 1:**
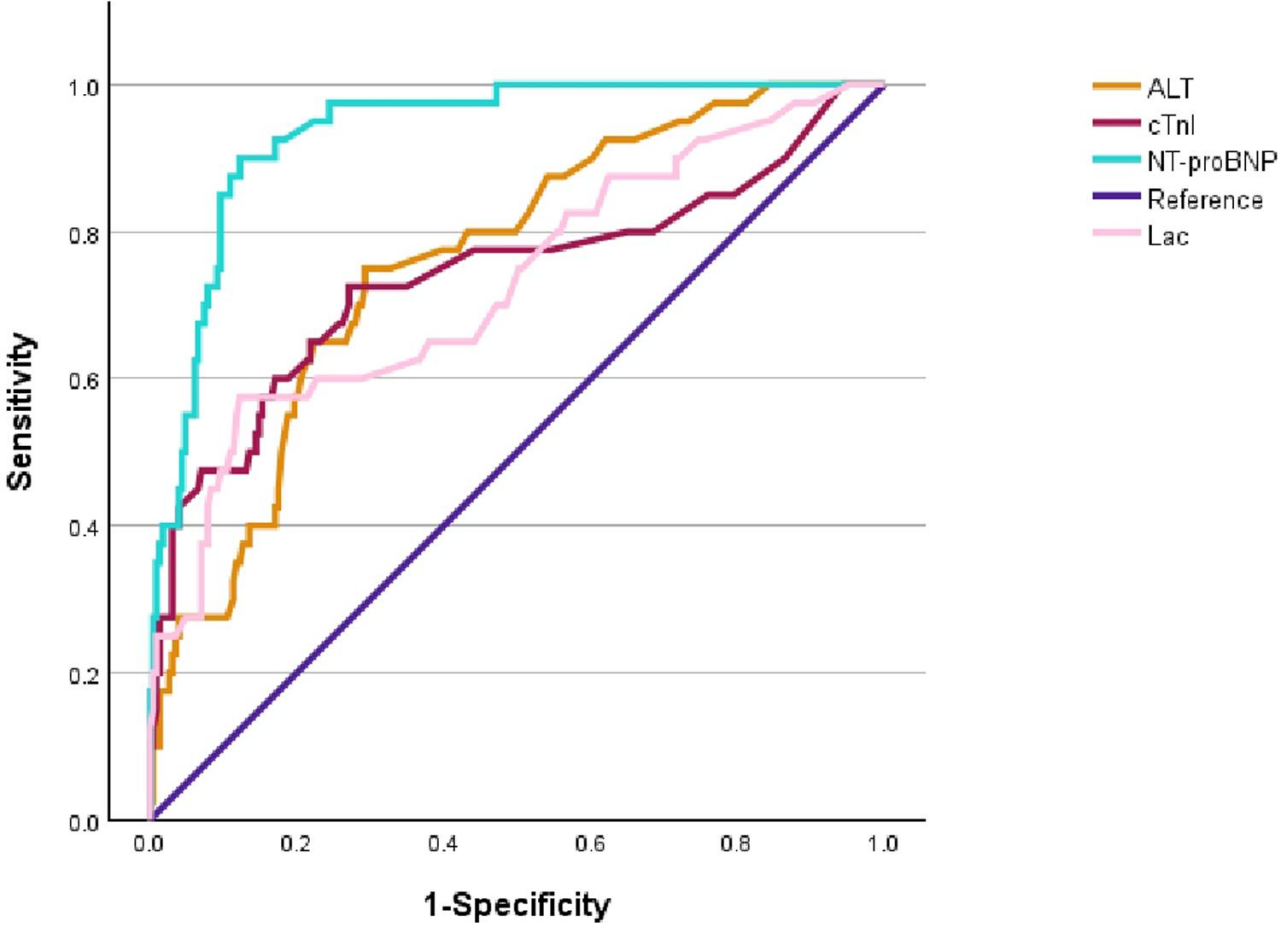
Receiver operating characteristic (ROC) curve analysis of early identification of fulminant myocarditis.

Logistic regression analysis suggested that hypotension, chest distress, Lac, ALT, cTnI and NT-proBNP were independent risk factors for fulminant myocarditis (Table 2). ROC curves revealed that NT-proBNP, cTnI, ALT, and Lac could predict independent risk factors for fulminant myocarditis. NT-proBNP had the strongest predictive value for risk factors, followed by ALT, cTnI and Lac (Figure 1, Table 3).

**Table 2:** Logistic regression analysis of early identification risk factors in the fulminant myocarditis.

Risk factors	B	OR	95% CI	*P*
Lac >3.6 mmol/L	1.180	3.255	1.063–9.968	0.039
NT-proBNP >2,000 pg/ml	3.004	20.161	5.659–71.825	<0.001
ALT >49 U/L	1.187	3.277	1.199–8.956	0.021
cTnI	1.350	3.859	1.513–9.843	0.005
Chest distress	2.179	8.841	1.969–39.694	0.004
Hypotension	1.505	4.506	1.380–14.711	0.013

**Table 3:** Analysis of coincidence rate, sensitivity and specificity of NT-pro BNP, ALT, cTnI and Lac in the independent risk factors for the fulminant myocarditis.

Variables	AUC (95% CI)	Cut-off level	Sensitivity (%)	Specificity (%)	*P*
NT-proBNP (pg/ml)	0.935 (0.902–0.968)	2,783.5	90	87.8	0
ALT (U/L)	0.759 (0.683–0.835)	34.5	75	70.7	0
cTnI (ug/ml)	0.739 (0.638–0.840)	0.2	72.5	72.9	0
Lac (mmol/L)	0.729 (0.636–0.822)	3.05	57.5	87.8	0

### Patient management

All patients received treatments, including IVIG (58.0%), corticosteroids (47.2%), vitamin C (69.9%), and vasoactive drugs (20.1%). However, ECMO was indicated for patients with severe heart failure, cardiac arrest and refractory shock, and some patients with combined renal failure received CRRT support. In the nonfulminant myocarditis group, the use of IVIG, corticosteroids, and vasoactive drugs by patients was significantly lower than that in the fulminant myocarditis group (Table 1).

ECMO was initiated in 19 patients, among whom 2 patients had temporary pacemakers installed before ECMO. 6 patients received CRRT due to renal failure. Notably, one patient with fulminant myocarditis was complicated because of also having Kawasaki disease (KD) (Table 4).

**Table 4:** Characteristics, treatment, and outcomes of ECMO in patients with the fulminant myocarditis.

Patient	Age, y	Sex	Symptoms	Temporary pacemaker	IVIG	Corticost-eroids	ECMO duration, h	CRRT	Outcome	LVEF at admission, %	LVEF at follow-up (1 year), %	Complications	Cause of death
1	6	F	Fever, chest pain, hypotension		Yes	Yes	520		Death	34	NA		Septic shock
2	7	F	Fever, hypotension		Yes	Yes	235	Yes	Recovery	38	68	Renal failure	
3	6	F	Fever, hypotension		Yes	Yes	24		Death	43	NA		Cardiac shock
4	4	F	Fever, chest distress		Yes	Yes	144		Recovery	24	68		
5	7	M	Fever, hypotension		Yes	Yes	216		Recovery	40	73		
6	12	F	Fever, hypotension		Yes	Yes	60	Yes	Death	12	NA	Renal failure	MODS
7	12	F	Fever		Yes	Yes	168	Yes	Recovery	23	68	Renal failure	
8	7	F	Fever, abdominal pain		Yes	Yes	113		Recovery	43	67		
9	6	M	Fever		Yes	Yes	603		Death	54	NA		Cardiac shock
10	12	F	Fever		Yes	Yes	311		Recovery	59	60		
11	9	F	Fever		Yes	Yes	228		Recovery	24	58	KD	
12	9	M	Hypotension	Yes	Yes	Yes	113		Death	30	NA		MODS
13	6	M	Fever, hypotension		Yes	Yes	305		Recovery	49	77		
14	17	M	Palpitation, hypotension				24		Death	48	NA		Cardiac shock
15	12	M	Fever, hypotension		Yes	Yes	152		Recovery	26	69		
16	10	F	Fever, hypotension		Yes	Yes	1,557	Yes	Death	24	NA	Renal failure	MODS
17	4	F	Hypotension		Yes	Yes	323		Recovery	22	51		
18	15	F	Chest distress	Yes	Yes	Yes	337	Yes	Recovery	47	60	Renal failure	
19	6	F	Fever, hypotension		Yes	Yes	253	Yes	Death	23	NA	Renal failure	Cardiac shock

ECMO, extracorporeal membrane oxygenation; CRRT, continuous renal replacement therapy; MODS, multiple organ dysfunction syndrom.

### Survival rate and follow-up

In this study, 8 patients died; the overall survival rate of patients with acute myocarditis was 97.03%. The survival rate of patients with fulminant myocarditis treated with ECMO was 57.89%. The causes of death were cardiac shock (*n* = 4), multiple organ dysfunction syndrome (*n* = 3), and septic shock (*n* = 1) (Table 4). Long-term follow-up of patients with fulminant myocarditis was performed. The median follow-up time was 3.5 yeas (range 8 months–9 years), and there were no late deaths or heart transplants. The follow-up method was echocardiography. There was no cardiac MR.

Among the 19 patients with fulminant myocarditis who required ECMO support, only one surviving patient exhibited delayed recovery of left ventricular function, however, by the third year of follow-up, the LVEF had still not returned to normal (LVEF < 55%). In contrast, all 21 patients with fulminant myocarditis who were managed without ECMO survived. Among the patients who survived from fulminant myocarditis, 31 of tnem had thier LVEF returned to normal 6 months after discharge.

## Discussion

The incidence rate of acute myocarditis in children in China remains unknown. However, research reports indicate that the annual incidence rate of acute myocarditis in Japanese children is approximately 3 per 1,000,000 (10). Owing to the insidious onset of myocarditis in some children, the true incidence and prevalence of myocarditis in children may be underestimated (11). The early clinical symptoms of acute myocarditis in children have no obvious specificity. In this study, the clinical manifestations of patients upon admission were primarily respiratory symptoms such as cough and shortness of breath, and digestive symptoms such as nausea, vomiting, and abdominal pain. These symptoms were more prominent than those typically associated with cardiovascular diseases, which often led to misdiagnosis or missed diagnosis. In the fulminant myocarditis group, fever and vomiting were more common at onset. Children with fulminant myocarditis often experience rapid symptom progression, which may include severe hemodynamic disturbances (12). This study revealed that children in the fulminant myocarditis group were more prone to hypotension than those in the nonfulminant myocarditis group.

Acute myocarditis lacks specific diagnostic methods, and myocardial injury markers are often used as reference indicators for the diagnosis of acute myocarditis. NT-proBNP is an indicator that reflects the left ventricular end-diastolic pressure and measures the filling pressure in the heart's main pump chamber (13). It is secreted by ventricular myocytes in response to volume or pressure overload to counteract the effects of the renin-angiotensin-aldosterone system and the sympathetic nervous system during heart failure (14). Therefore, NT-proBNP can serve as a key prognostic factor for evaluating disease severity and patient outcomes (15). cTn is currently the marker with the highest specificity for diagnosing myocardial injury that has been discovered. cTnI is released into the blood in the early stage of myocardial injury, begins to increase at 3–12 h and reaches its peak at 12–24 h; the detection window lasts for 7–14 days. Although elevated cTnI is a good biomarker for myocarditis, higher levels are not associated with the severity of the disease. In a multicenter review of pediatric myocarditis, the cTnI level in patients with mild ventricular dysfunction was significantly higher than that in patients with moderate or severe ventricular dysfunction (16). Sachdeva et al. reported no association between cTnI levels and outcomes in a single-center retrospective study, and late enhancement on CMR was a risk factor for poor prognosis in the early or late stages of myocarditis (17). Therefore, elevated cTnI levels should be considered an important indicator for the diagnosis of myocarditis, but should not be used to classify its severity or predict prognosis. Lactic acid, a product of anaerobic glycolysis, plays a crucial role in metabolism and exercise. It is a common laboratory indicator that shows the severity of different diseases (18). Lactate has become useful as a risk factor for predicting patient mortality and an important prognostic indicator in intensive care settings (19, 20). In this study, almost half of the patients with acute myocarditis presented elevated transaminase levels, which may be associated with shock and congestive heart failure or could be caused by the same viral triggers responsible for myocarditis. A retrospective single-center pediatric study revealed that serum AST levels were elevated in children with myocarditis (20). However, in our study, we found that the increase in ALT was more obvious. Previous studies have reported that serum markers reflecting the inflammatory state of the heart and the function of terminal organs, including troponin-I, liver enzymes, creatinine, and serum lactic acid, are correlated with the course of the disease (21, 22). This study suggested that NT-proBNP, ALT, cTnI, and Lac can serve as predictors for the early diagnosis of fulminant myocarditis. The optimal predictive values for these markers are 2,783.5 pg/ml for NT-proBNP, 34.5 U/L for ALT, 0.2 µg/ml for cTnI, and 3.05 mmol/L for Lac. In previous studies, Moises et al. reported that NT-proBNP can predict risk factors for the early identification of fulminant myocarditis. The test AUC was 0.931, the optimal cutoff value was 2,000 ng/ml, the sensitivity was 90%, and the specificity was 81% (23). Lee et al. reported that the lactate level before ECMO can predict risk factors for cardiac arrest death, with an AUC of 0.848, an optimal cutoff value of 79.8 mg/dl, a sensitivity of 70%, and a specificity of 91% (4). Relevant studies on cTnI have been conducted in adults with myocarditis, indicating that elevated cTnI has a very low sensitivity (34%) for the entire myocardial group, but the specificity is satisfactory (89%) (24). Freedman also reported that AST measurement might be a useful laboratory-assisted means, reporting sensitivity in 85% of patients with myocarditis. The median AST value in the determined group was 66 U/L, and that in the possible group was 116 U/L (20). However, no reports related to ALT levels have been published.

Acute myocarditis can damage the myocardium, and surrounding tissues, leading to ventricular systolic dysfunction. It can also simultaneously affect the cardiac conduction system, causing changes in automaticity and conductivity (25). In this study, the fulminant myocarditis group was more prone to supraventricular tachycardia, ventricular tachycardia, and III° atrioventricular block than the nonfulminant myocarditis group (57.5%). Teele et al. previously reported (26) that approximately 55% (11/20) of pediatric patients with fulminant myocarditis have ventricular tachycardia and Ⅲ° atrioventricular block. This finding is consistent with previous research results. Echocardiography is an important tool for evaluating cardiac function. As the disease progresses, patients may exhibit abnormal left ventricular systolic function, a decreased ejection fraction, reduced ventricular wall motion, valvular regurgitation, and pericardial effusion (27). In this study, statistically significant differences were observed in LVEF, LVFS, MR, TR, and pericardial effusion between the fulminant myocarditis group and the nonfulminant myocarditis group.

Currently, there are no specific drugs for the treatment of myocarditis. The main treatment, including corticosteroids and IVIG, is symptomatic and only supportive. A recent retrospective analysis of 112 pediatric patients with myocarditis indicated that the combination of IVIG and high-dose corticosteroids is beneficial for improving left ventricular systolic function, with no obvious serious adverse events reported (28). However, currently, there is still controversy regarding the use of both treatments (29). In this study, the utilization rates of early IVIG and corticosteroids in the nonfulminant myocarditis group were 51.1% and 28.4%, respectively. In contrast, in the fulminant myocarditis group, IVIG and corticosteroids were administered to all patients except one, and they achieved noticeable effects. Some patients with fulminant myocarditis experience rapid disease progression. Even with timely drug treatment, circulation challenges remain difficult to correct in some patients, necessitating ECMO support to maintain circulation and allow time for myocardial recovery. The survival rate of patients with pediatric fulminant myocarditis treated with ECMO ranges from 64.3% to 83.3% (30, 31). Among patients with fulminant myocarditis, 19 received ECMO treatment, and there were a total of 8 deaths. The ECMO survival rate was 57.9%, slightly lower than the rates reported in the previous literature. This may be attributed to the relatively recent development and potentially limited experience with ECMO technology. At the onset of the disease, 2 patients were initially fitted with temporary pacemakers to support III° atrioventricular block. However, as cardiac output and tissue perfusion could not be effectively restored, ECMO circulatory assistance was eventually initiated. Additionally, 6 patients were treated with CRRT simultaneously, but due to the severity of their conditions, 3 of them still died.

**Figure 2:**
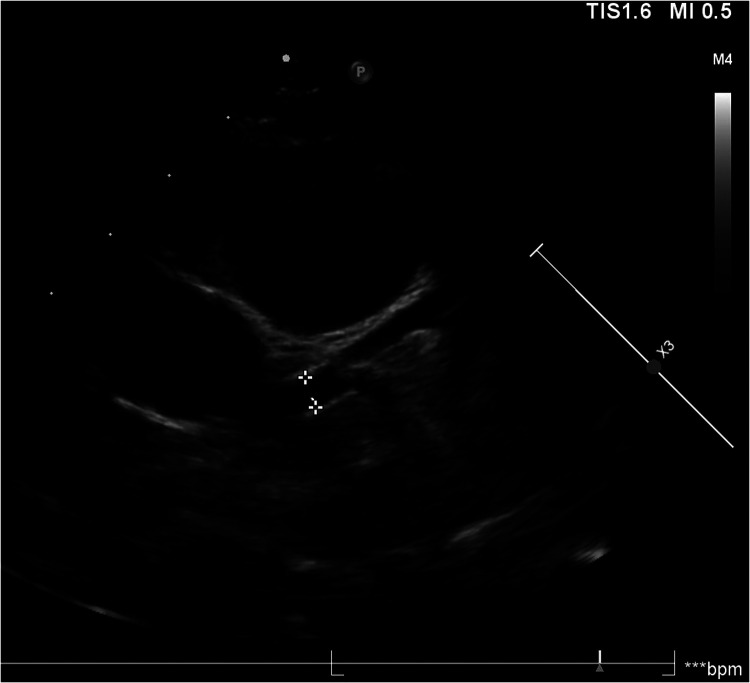
Images of small aneurysms in patient with concomitant kawasaki disease. On the 20th day of the disease course, the patient presented with fever, generalized diffuse rash, red and chapped lips, bayberry tongue, bilateral cervical lymph node enlargement and swelling of hands and feet, and diagnosed with KD. On the second day after the diagnosis of KD, echocardiography revealed that the left coronary artery small aneurysms (*Z* = 2.53).

Among patients with fulminant myocarditis, one patient had concomitant Kawasaki disease (KD), which is particularly noteworthy. After the patient was diagnosed with fulminant myocarditis, IVIG and corticosteroid therapy were immediately administered. At 15 days after ECMO withdrawal, the patient developed a fever, a diffuse red miliary rash on the trunk, red and chapped lips, swelling of the hands and feet, and enlarged cervical lymph nodes. Echocardiography revealed the formation of small aneurysms in the left coronary artery (Figure 2). Therefore, the diagnosis of KD was confirmed according to diagnostic criteria. Myocarditis often occurs in patients with KD. A previous study revealed that the incidence of myocarditis in individuals with KD can reach 50% (32). Generally, myocarditis in KD patients is self-limiting or well managed with IVIG. This type of myocarditis is mostly mild and is accompanied by subclinical left ventricular dysfunction. However, in a small number of patients, left ventricular dysfunction can be observed, leading to congestive heart failure or cardiogenic shock (33). However, based on the patient's medical history and the course of the disease, the pathogenesis remains unclear.

Most patients with myocarditis have a good prognosis and can be cured. However, approximately 16% of patients are at increased risk of developing cardiomyopathy during the follow-up period (23, 33, 34). Studies have shown that patients with acute myocarditis have a good long-term prognosis if effective treatment is given in the early stage of the acute phase (35). In this study, long-term follow-up of patients with fulminant myocarditis was conducted by this center. The median follow-up duration was 3.5 years (range 8 months–9 years), during which no late deaths or heart-transplant procedures occurred. The functional recovery mainly occurs in the first few weeks of the disease, which means that rapid therapeutic intervention is necessary. Surveillance relied on serial echocardiography. Cardiac MRI was not performed. However, during the subsequent follow-up visits, cardiac MR examinations will be gradually conducted. This has been listed by us as one of the directions for further research in the future. In this study, the LVEF of one patient still had not returned to normal (LVEF < 55%) at the 3rd year of follow-up, while the EF values of the remaining patients returned to normal within half a year after discharge.

This study has several limitations. Acute myocarditis was diagnosed clinically without confirmation through myocardial biopsy. Furthermore, the incidence of fulminant myocarditis is low, resulting in a small sample size. This may introduce certain biases into the results, and a larger sample size with a multicenter design is needed for further research. Additionally, owing to incomplete follow-up data, it is not possible to evaluate the long-term outcomes of patients with acute myocarditis.

## Conclusions

Early identification of fulminant myocarditis, coupled with timely administration of effective treatment measures, can significantly improve patient prognosis. In this study, the survival rate of patients with acute myocarditis reached 100%, whereas that of patients with fulminant myocarditis was 80%. This study focused on the follow-up of patients with fulminant myocarditis. During the follow-up period, no deaths were reported. However, the LVEF of one patient had not yet fully normalized. NT-proBNP, cTnI, ALT, and Lac can serve as predictive factors for the early identification of fulminant myocarditis. These findings emphasize the importance of early identification and timely diagnosis in improving the overall prognosis of myocarditis patients.

## Data Availability

The original contributions presented in the study are included in the article/Supplementary Material, further inquiries can be directed to the corresponding author.
